# Characterization of a new natural fiber extracted from *Corypha taliera* fruit

**DOI:** 10.1038/s41598-021-87128-8

**Published:** 2021-04-07

**Authors:** Taslima Ahmed Tamanna, Shah Alimuzzaman Belal, Mohammad Abul Hasan Shibly, Ayub Nabi Khan

**Affiliations:** 1 Department of Fabric Engineering, Bangladesh University of Textiles, Dhaka, 1208 Bangladesh; 2 Department of Textile Engineering, BGMEA University of Fashion and Technology, Dhaka, 1230 Bangladesh; 3 Department of Textile Engineering, National Institute of Textile Engineering and Research, Dhaka, 1350 Bangladesh

**Keywords:** Environmental sciences, Engineering, Materials science

## Abstract

This study deals with the determination of new natural fibers extracted from the *Corypha taliera fruit* (CTF) and its characteristics were reported for the potential alternative of harmful synthetic fiber. The physical, chemical, mechanical, thermal, and morphological characteristics were investigated for CTF fibers. X-ray diffraction and chemical composition characterization ensured a higher amount of cellulose (55.1 wt%) content and crystallinity (62.5%) in the CTF fiber. The FTIR analysis ensured the different functional groups of cellulose, hemicellulose, and lignin present in the fiber. The Scherrer’s equation was used to determine crystallite size 1.45 nm. The mean diameter, specific density, and linear density of the CTF fiber were found (average) 131 μm, 0.86 g/cc, and 43 Tex, respectively. The maximum tensile strength was obtained 53.55 MPa for GL 20 mm and Young’s modulus 572.21 MPa for GL 30 mm. The required energy at break was recorded during the tensile strength experiment from the tensile strength tester and the average values for GL 20 mm and GL 30 mm are 0.05381 J and 0.08968 J, respectively. The thermal analysis ensured the thermal sustainability of CTF fiber up to 230 °C. Entirely the aforementioned outcomes ensured that the new CTF fiber is the expected reinforcement to the fiber-reinforced composite materials.

## Introduction

In the last few decades, crop surplus and natural resources have been considered as an alternative of synthetic fiber reinforced polymer-based substance for industrial components such as automobiles, aviation, marine, textiles, and domestic applications^1,2^. Natural fibers show attractive properties over synthetic fibers for instance low weight and cost, biodegradable, and availability in the environment^3,4^.Due to the awareness of environmental protection industrialists, researchers, and academicians are developing sustainable composite materials. Natural fiber-based manufacturing plants can control the emission of hazardous chemical and non-degradable waste generation during the manufacturing over the synthetic fibers-based manufacturing plant^5^. Natural fiber-reinforced composites have distinctive advantages with compare to synthetic materials and are highly influenced by the green environments, such as high specific properties, strength, better stiffness, lightweight, biodegradability, thermal insulation, abundance, low cost, nonabrasive nature, nontoxicity, and so forth^6–8^. The natural fiber-based composite’s quality depends on mainly reinforcement properties and matrix attachment. The complete bio-based natural composites are made with a combination of natural fiber and natural-based resin. These composites do not turn out allergic and irritation behavior in the human body. Although natural fiber contains commonly hemicellulose, cellulose, lignin, pectin, wax, and moisture, but the characteristics of those fibers completely depend on the growing environment of plants, type and maturity of the plant, fiber extraction method, and fiber extraction part of the tree^9,10^. The natural reinforced materials have been taken out from different portions of the plant for example roots, seeds, stems, leaves, bark, and fruits, etc. and these are the utmost important sources of cellulosic fibers^11,12^. In this research work, the *Corypha taliera* fibers are extracted from fruit’s seed, and this plant grown in the Indian subcontinent^13^. *Corypha taliera* plant is one of the palm species and can provide fruit juice and fiber, and leave fiber^14^.

A large number of researches have been carried out with the natural fibers characterizations for example Cotton, Jute, Ramie, Flax, Coir, Rice husk, Kenaf, Sugar cane, Grass, *Pineapple leaf* fibers. Among all-natural fibers, only natural cellulosic fiber composite provides environmental facilities compared to disposability and raw material utilization^15^. Huge amount of natural cellulosic fibers are required to alternate synthetic fibers and the current production of natural fibers does not satisfy industry demand. Such novel potential natural fibers are to be prescribed with easy and cost-effective fiber extraction methods. New potential fibers are to be investigated to determine the chemical, thermal, mechanical, and physical properties^16^. The seed and bark fibers as an alternative source for fiber-reinforced composite were investigated in the research^17^. The study results illustrate that the bark fibers had higher cellulose and lower lignin and extracted fibers compared to the seed fibers.

In this research work, the fiber of the seed, *Corypha taliera* ripe fruit’s fiber, which is belongs to the family of the palm tree and was selected for fiber removal and prospective estimation as reinforcement in composite. The CTF covers cellulosic semisolid flush which is reinforced by the fiber. This fruit juice is used as a food beverage and the fibers are left as waste. The CTF fibers are eco-friendly, inexpensive, available in nature, renewable and therefore the determination of the fiber potential characteristics to the technical arena is essential. The main objective of the current research was therefore to investigate the physical, mechanical, and chemical properties of CTF fibers. The CTF fibers were not characterized extensively until today to measure its potentiality to replace artificial fiber for polymer composite reinforcement. Henceforward, this study results can be utilized by the technologist, academician, chemist, and scientist for further applied research.

## Material and experiments

### Extraction of CTF fibers

**Figure 1:**
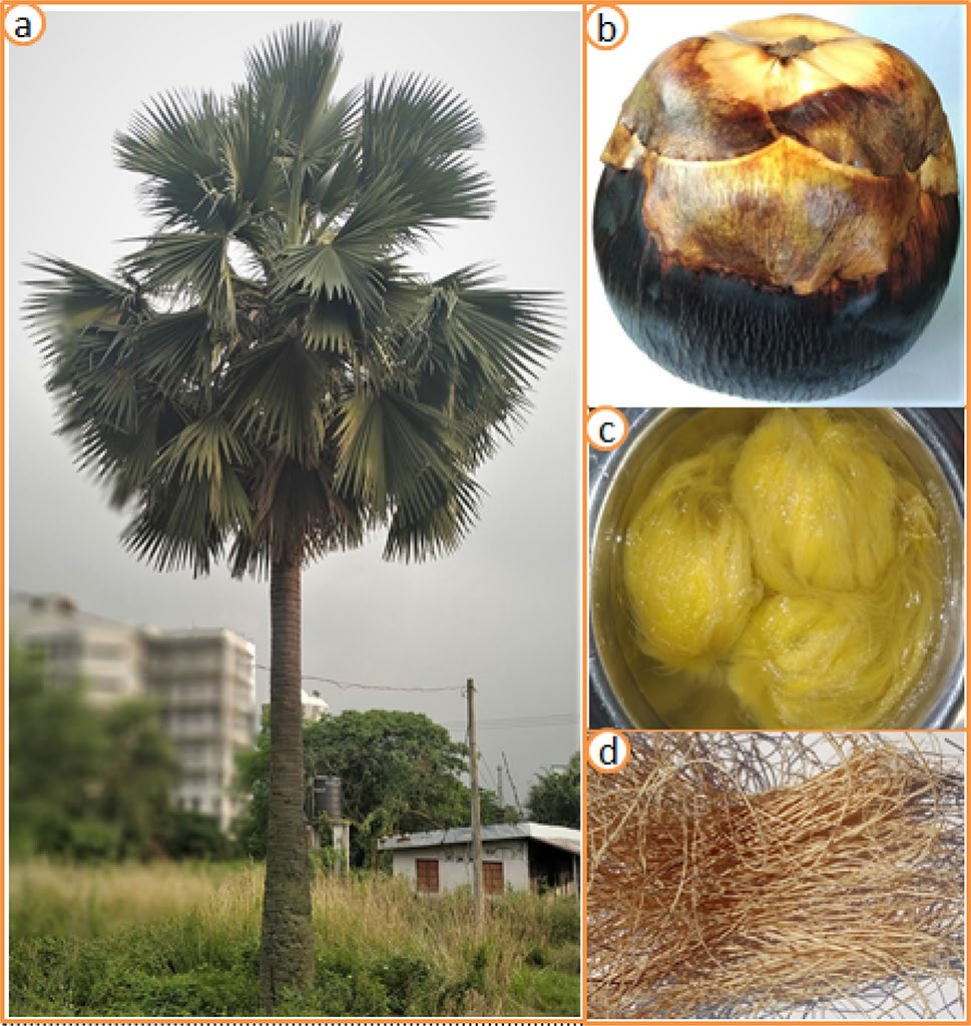
*Corypha taliera fruit* fiber extraction: (**a**) *Corypha taliera* plant; (**b**) *Corypha taliera* fruit; (**c**) Fruit seeds immersed into water; (**d**) Extracted dry fibers.

In this study, the CTF’s fiber was used as raw material and obtained from the *Corypha taliera* ripe fruit’s seed. CTF was collected from Gazipur district, Bangladesh. The *Corypha taliera* tree is a species of palm and originally available in Myanmar, Bangladesh, and India. This palm tree was discovered by William Roxburgh in 1819 and it’s locally called Talipalm^18^. The *Coryphe taliera* seeds were peeled off from the fruit and immersed in water for 15 min. After that, the immersed seeds were washed thoroughly by hand to remove the juice from the seeds. The CTF fibers were cut out from the seed and washed again under the temperature of 50 °C for 2 h. In this way, fiber released excessive juice from the fiber surface and thoroughly cleaned with the help of fresh water. Followed by, the washed fibers were dried in direct sunlight for about the two-day of time. The tree, fruit, seeds from which the fibers got collected, and the image of the dried fiber are shown in Fig. 1a–d respectively.

### Physical characterization

The CTF fibers were equilibrated under standard conditions, i.e. 20 °C temperature and 65% RH for 48 h^19^. To measure the fiber diameter, 3 random places of 100 fibers were measured by an optical microscope, and longitudinal direction images were taken from distinct fiber samples. Finally, the mean diameter of the CTF fiber was calculated using the software “Image Pro Plus”^20,21^. The count of CTFF was determined in terms of linear density according to ASTM D 1577–92 in Tex system^22^. Twenty-five distinctive fibers were taken to determine the average count. The density of CTF’s fiber was assessed using the pycnometer setup where a known toluene liquid density was used to identify fiber density. Then, the density of CTF’s fiber was determined using the following equation^23^.

1 $$ \rho_{CTFF} = \frac{{\left( {m_{2} - m_{1} } \right)}}{{\left( {m_{3} - m_{1} } \right)\left( {m_{4} - m_{2} } \right)}} \times \rho_{t} $$

where $\rho_{CTFF}$ express the density of CTF fibers (g/cm^3^) and $\rho_{t}$, $m_{1}$, $m_{2}$, $m_{3}$, $m_{4}$ are density of toluene, mass of the empty pycnometer, mass of the pycnometer filled with chopped CTF fibers, mass of the pycnometer filled with toluene, and mass of the pycnometer filled with chopped fibers and toluene in kg, respectively.

### Chemical characterization

#### Chemical composition

The chemical compositions of *Corypha taliera fruit* (CTF) fiber were performed according to TAPPI standard methods. The determination of percentage of lignin content was made by the following of TAPPI T211 om-83^24^. To determine the holocellulose and cellulose content in the fiber, the TAPPI t249 and TAPPI T203 om-93 methods were followed, respectively^25,26^. The hemicellulose was determined by the following equation.

2 $$ \%\, Hemicellulose = \% {\text{\,Holocellulose}} - {\text{\% Cellulose}} $$

The extractive content of the fiber was estimated in accordance with TAPPI standard T204 om-88 methods^27^.

#### Moisture content

The moisture content percentage was measured by using the ASTM D 2495 method. A 5-g sample was taken under the standard atmosphere conditions for testing fibers at 20 °C and 65% RH. The weighted fiber samples were placed in an air oven at a constant temperature at 105 °C. After 15 min interval sample weight was measured until there 0.1% less change in sample mass between successive weighing^28^. The difference between standard conditioning weight and oven-dry weight yields moisture content in the CTF fibers. The moisture content percentage of fiber was calculated by using the following equation^29,30^.

3 $$ M\% = \frac{{W_{1} - W_{2} }}{{W_{1} }} \times 100 $$

where M% denotes the percentage of moisture content, W_1_ and W_2_ are the fiber weight before woven dry and after oven dry in grams.

### Spectroscopic characterization (FTIR)

The functional compound of the fiber was measured by using the Fourier Transform Infrared (FTIR) Spectrophotometer (FT-IR 8400S, Shimadzu Corporation, Japan)^31,32^. To measure the infrared of CTF fiber, the fibers were crushed and blended with potassium bromide (KBr) due to the transparent nature of KBr. The scan rate of FTIR spectrometer was 32 per min and resolution of 2 per cm in the wave number region range of 400–4000 cm−^1^ at a room temperature of 30 °C and RH of 65% was documented in absorbance mode as a function of the wave.

### X-ray diffraction (XRD)

The crystallinity index of the CTF fiber specimen was studied using XRD^33^. The degree of structural arrangement is determined through the crystallinity index whose amount is paramount important because it influences the alkali treatment and mechanical properties of natural cellulosic fibers^23^. The study was carried out on BRUKER AXS Diffractometer D8, Germany; using Cu Kα radiation at operating condition was V = 40 kV, I = 40 mA. The X-Ray detector offered in the diffractometer was used to scan the diffracted X-Ray between 0° and 100° (2θ) at a scan speed 3°/min in steps of 0.02°. The crystallinity index (CI) of CTF fiber was then determined using empirical methods^34^, as presented in Eq. (4).

4 $$ CI\left( \% \right) = \frac{{\left( {I_{200} - I_{am} } \right)}}{{I_{200} }} \times 100 $$

where, $I_{200}$ is the maximum intensity of the peak between 22° and 23° for the crystalline area at a 2θ angle, and $I_{am}$ is the minimum intensity of an amorphous region between 18° and 19° at a 2θ angle^35^. The crystallite size (CS) of CTF fiber was determined by the Scherrer’s equation as given below:

5 $$ CS = \frac{{K{{\lambda }}}}{\beta cos\theta } $$

where $K{ }$ denotes Scherrer’s constant, whose value is 0.89,${\text{ and}},\, {\lambda }$ $\beta$, $\theta$ are wavelength of the radiation, full-width at half-maximum, Bragg angle, respectively.

### Mechanical properties

The tensile strength and Young’s modulus, elongation at break, and energy at break of the CTF fiber were enumerated according to ASTM D 3822–07 at room temperature using Hounsfield, H10KS, testing machine, UK, with crosshead speed of 10 mm/min and two different fiber gauge length, 20 and 30 mm, at 68 ± 3% relative humidity^16,36^. Twenty CTF fibers were tested in each gauge length, and the average test results were reported. To measure the force, a known load cell 1.0 KN was used in this experiment^37^. The fiber diameter and tensile strength is analyzed statistically by Weibull distribution using Minitab 18 software. The tensile strength and Young’s modulus were calculated by Eq. (6).

6 $$ \sigma = \frac{{F_{b} }}{{S_{0} }} $$

where $\sigma$ represents the tensile strength and $F_{b}$ and $S_{0}$ denotes max force at fiber break and the cross-sectional area of fiber respectively. Young’s modulus and elongation at break were calculated from the test data.

### Thermal analysis

#### Thermogravimetric analysis

Thermal Gravimetric Analysis (TGA) was carried out to measure the thermal stability of the CTF fibers by thermal analyzer SDT650, synchronized TGA, and DSC device of TA Instruments, USA. TGA analysis is a paramount important one for the experiment of thermal permanence of the constituents of the natural fiber considers the functioning temperature limit of the composite made with similar fibers^38^. For the experimental process, 10 mg of CTF fiber were used. TGA and DSC experiments were performed in nitrogen atmosphere conditions from room temperature to 600 °C at a constant heating and flow rate 10 °C/minute and 30 ml/minute, respectively^39^.

According to Broido’s equation (Eq. 7), the Kinetic Activation Energy (Ea) of the CTF fiber was found. The term kinetic activation energy is the least amount of energy required to degrade the fiber.

7 $$ {\text{In}}\left[ {In\left( \frac{1}{y} \right)} \right] = - \left( \frac{Ea}{R} \right)\left[ {\left( \frac{1}{T} \right) + K} \right] $$

where, y denotes normalized weight (w_t_/w_i_), w_t_ denotes the weight of the sample at any time t, w_i_ represents the initial weight of the sample, T denotes the temperature at the considered time and R represents universal gas constant (8.32 kJ/mol-k).

#### Differential scanning calorimeter analysis

The analysis of DSC was conducted for supporting the thermogravimetric analysis. The 10 mg mass was kept inside the pans and sealed tightly. After that, the sealed pan was placed inside the calorimeter and heated of inert gas nitrogen up to 45 °C. Significant temperatures of melting peak readings were recorded at 10 °C/min heating rate^40^.

### Morphology study

#### Scanning electron microscopy (SEM)

The surface morphologies of the CTF fiber were studied by Scanning Electronic Microscopic (SEM) (ZEISS EVO 18) at an operating voltage 10 kV. Before the experiment, the samples were coated with gold in a vacuum atmosphere to improve the conductivity of fiber.

#### Energy dispersive X-ray spectroscopy (EDX)

EDX is a common method used to identify elements (for instant Carbon, Oxygen, and Nitrogen, etc.) of natural fiber. The elemental presence of CTF fiber was determined by EDX (TEAM™ EDS), which is equipped with the SEM.

## Results and discussions

### Physical characterization

**Figure 2:**
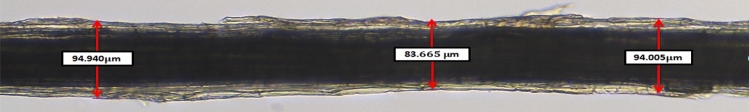
Optical microscopic image of *Corypha taliera fruit* fiber.

Appropriate fiber diameter measurement of any natural fiber is quite tough due to uneven thickness. With the change of environment and growing condition natural fiber does not have a uniform diameter throughout its entire length^15^. The diameter range of CTF fibers was 45 to 548 μm, and the mean diameter was determined to be 131 μm. Figure 2 shows the diameter measurement and microscopic view of a fiber through the Image Pro Plus software. The density value of the CTF fiber was measured to be 0.86 g/cc. The density of natural fiber was given similar importance in case of mechanical characteristics investigation due to highlight these fibers from artificial fibers^41^. The density of the CTF fiber is closer to the *Phaseolus vulgaris*, *Sansevieria ehrenbergii*, *Oil palm*, and Date fiber as given in Table 1. The single fiber fineness of the CTF fiber is 43 tex or 387denier. The comparison of chemical and physical properties of CTF fibers with other natural fibers is presented in Table 1.

**Table 1:** Comparison table of physical and chemical properties of CTF fibers with other natural fibers.

Fiber	Physical properties		Chemical properties			
	Diameter ($\upmu$m)	Density (g/cc)	Cellulose (%)	Hemi-cellulose (%)	Lignin (%)	Moisture (%)
*CTF fiber*	45–548	0.86	55.1	21.78	17.6	7.1
*Borassus fruit fiber*^32^	203.12–287.23	1.256	68.94	14.03	5.37	6.83
*Ficus religiosa*^36^	25.62	1.246	55.58	13.86	10.13	9.33
*Jute*^37,42^	40–350	1.46	64.4	12	11.8	1.1
*Ramie*^42^	50	1.5	68.6	13.1	0.6	8.0
*Flax*^42^	–	1.5	71.0	18.6	2.2	10.0
*Phaseolus vulgaris*^15^	53.56	0.852	62.17	7.04	9.13	6.1
*Kenaf*^37^	65–71	1.4	45–57	8–13	21.5	6–12
*Sisal*^37^	50–300	1.5	60–78	10–14.2	8–14	10–22
*Cotton*^30,37^		1.6	82.7	5.7	–	7.85–8.5
*Ferula communis*^43^	90–300	1.24	53.3	8.5	1.4	24.8
*Acacia leucopholea bark*^44^	168.5	1.385	68.09	13.6	17.73	8.83
*Bamboo*^30,37^	240–330	9.1	73.83	12.49	10.15	9.16
*Coconut tree leaf sheath*^37^	140–990	1.2	27	14	27.7	4.7
*Sansevieria ehrenbergii*^30^	10–250	0.887	80	11.25	7.8	10.55
*Date*^45^	155–250	0.99	–	–	–	10.67
*Coccinia grandis stem*^46^	543–621	1.5175	63.22	–	24.42	9.14
*Oil palm*^30,47^	150–500	0.7	65	10.12	17.5	–
*Coconut/Coir*^30,48^	100–450	1.15	44.2	12.1	32.8	11.36
*Pineapple leaf*^48^	53–62	1.32	73.4	7.1	10.5	–
*Banana stem*^30,48,49^	60–250	1.35	63.9	1.3	18.6	10.71

### Chemical characterization

The chemical composition of CTF fiber, some palm fibers, and other available natural fibers are presented in Table 1. Extractive (wax, fat, gum and pectin) was lowest in CTF fiber (1.3%) compared to other fibers; Coir 6.4%, *Pineapple leaf* 5.5%, and *Banana stem* 10.6%^30,48,49^. High extractives are considered as the important parameters in preventing the formation of microorganisms in the beginning periods of cloning^49^. In contrast, low extractives will increase the hygroscopic property of the fiber. This property influences minimum impact on chemical and mechanical characterization of composite materials.

The main component of CTF fiber shows holocellulose (76.88%) and cellulose content (55.1 wt%). Amount of cellulose in natural fibers is considered as the principal component which contributes to increase tensile strength, stability, stiffness, and resistance to hydrolysis as well as economic production of fibers for several uses^50^. Cellulose content of the CTF fibers is 1.8, 28.1, and 10.9% greater than the *Ferula communis*, *Coconut tree leaf*, and Coir fibers. The CTF fibers also contain 21.78% hemicellulose, 21.6% lignin, and 7.1% moisture. The hemicellulose is more compared to all-natural fibers in Table 1. The amount of lignin in fibers influences the structure, rigidity, and morphology and its value is less than *Kenaf*, *Accia leucopholea*, *Coconut tree leaf*, *Coccinia grandis stem*, and Coir fibers, and greater than Jute, Sisal, Cotton, Bamboo, and some others fibers. The content of lignin in CTF fibers is almost similar to *Oil palm* fiber (17.5%). The moisture percentage of the CTF fiber is comparable to natural fibers such as *Borassus fruit*, Ramie, Kenaf*, Phaseolus vulgaris* and other fibers.

### Spectroscopic characterization (FTIR)

**Figure 3:**
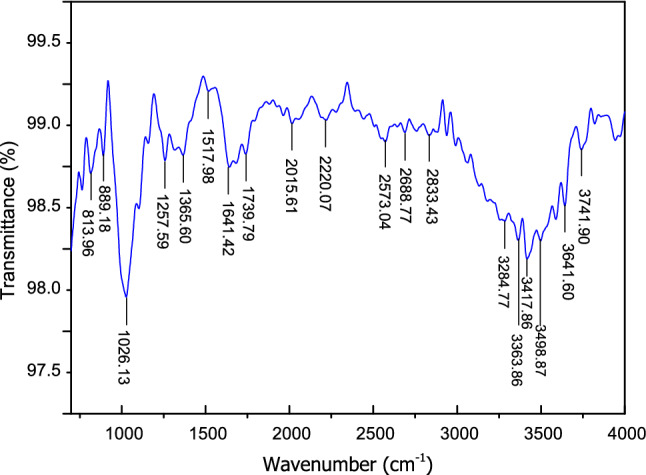
FTIR spectra of a CTF fiber.

The FTIR analysis of CTF fiber was performed to identify the presence of the chemical compounds in the raw fiber. Figure 3 shows the FTIR spectrum of wave number 700 to 4000 cm^−1^ of CTF fiber. The absorption bands show different chemical functional groups of lignocellulose fiber components, for example, hemicellulose, cellulose, and lignin with principal elements such as phenolic hydroxyl, alkenes, aromatic groups, and $\beta$-glucose linkages^51^. The absorbance peak at around 813.96 cm^−1^ indicates $\beta$-glycoside linkages of cellulose^15^, and 889.18 and 1517.98 cm^−1^ indicates stretching of the aromatic group such as in lignin^52^. The characteristic peaks at about 1026.13 cm^−1^ correspond to the C–O and O–H stretching vibration, which includes polysaccharide in cellulose and the absorbance peak at approximately 1257.59 cm^−1^ and 1365.6 cm^−1^ represents the presence of C–O stretching vibration of acetyl groups in lignin, hemicellulose and bending vibration of C–H, respectively^53^. The prominent peaks at 1641.42 cm^−1^ and 1739.79 cm^−1^ was assigned to C=O stretching vibration for acetyl groups in lignin and hemicelluloses^16,54^. An observable peak nearing 2015.61 cm^−1^ and 2220.07 cm^−1^ correspond to the C≡N and C≡C, and, and 2573.09 cm^−1^ corresponds to the S–H stretching. The peaks at 2688.77 cm^−1^ and 2833.43 cm^−1^ indicate C–H stretching vibration of CH_2_ and CH in cellulose and hemicellulose components^44^. The broad transmittance in the FTIR plot at 3284.77 cm^−1^, 3363.86 cm^−1^, 3417.86 cm^−1^, 3498.87 cm^−1^, 3641.9 cm^−1^, and 3741.9 cm^−1^ are assigned to the vibration of elongation of the O–H group. The presence of an O–H group can be explained that the O–H is associated with hydrogen bonding with the carboxyl of the fatty acids on the CTF fiber surface^15,55^.

### X-ray diffraction (XRD)

**Figure 4:**
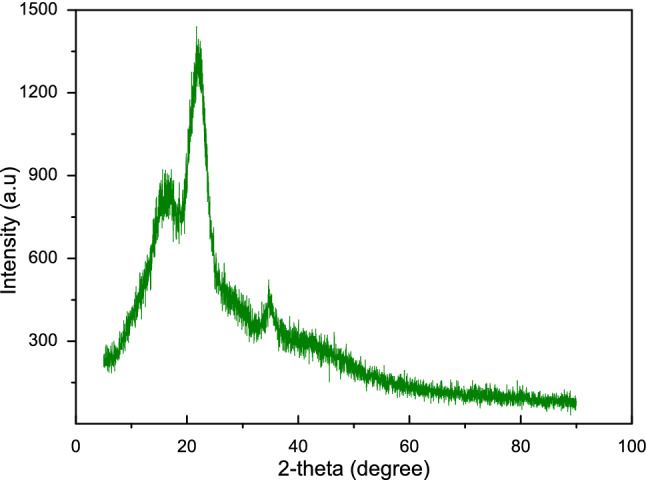
X-ray diffraction patterns of CTF fibers.

The X-ray diffraction patterns of CTF fiber is shown in Fig. 4. The spectrum shows three major diffraction peaks at 2θ angles of approximately 16.341°, 22.13°, and 35.12°, corresponding to (200), (110), and (004) lattice planes^42,56^. The first two main peaks are the characteristic peaks of cellulose I and cellulose IV^57^. The cellulose crystallinity index (CI) was determined as 62.5%, which is higher than that of *Grewia tilifolia* (41.7%), *Oil palm fruit* (34.1%), Palm fiber (19.9%), 7 Coconut fiber ((19.9%), 7, Cotton (60%), *Tamarindus indica fruit* fibers (55%), *Areca fruit husk* (55.5%). Besides, the CI is lower than that of Sisal (71%), Jute (71%), Hemp (88%) and Flax fiber (80%)^16,36,42,46,58,59^. The crystallite size was estimated from the spectrum with the aid of the Scherer’s equation, and the value is 1.45 nm, which is lower than *Ficus religiosa* (5.18), Cotton (5.5 nm), flax (2.8 nm), *Juncus effuses* (3.6 nm), *Tamarindus indica fruit* fibers (5.73 nm)^36,58,59^. The crystallite size of CTF fiber (1.45 nm) minimizes water absorption and chemical reactivity when reinforced in a matrix medium^46^.

### Mechanical properties

**Figure 5:**
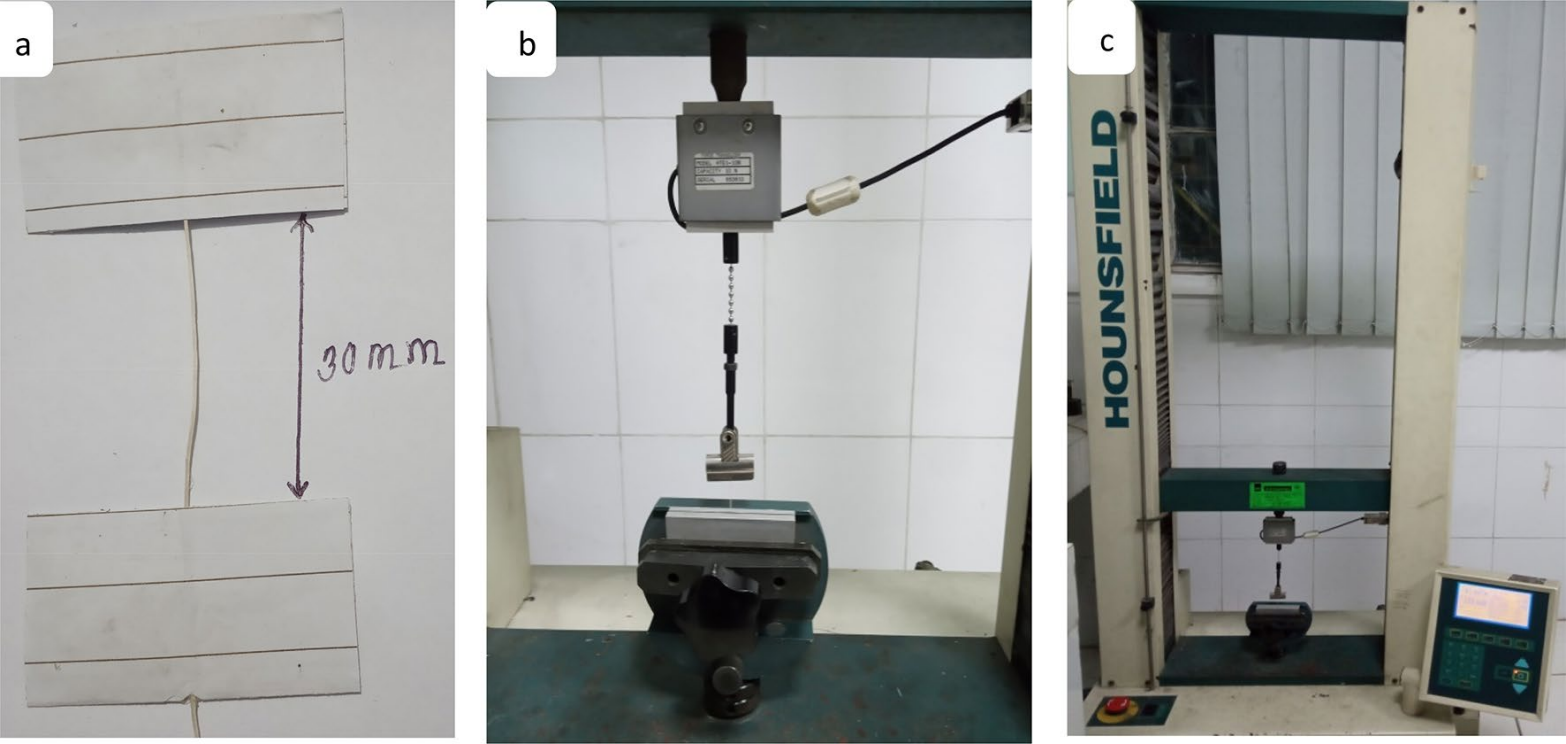
Images of single fiber tensile strength test (**a**) Sample preparation, (**b**) Sample setting with jaw, (**c**) Universal Testing machine.

**Figure 6:**
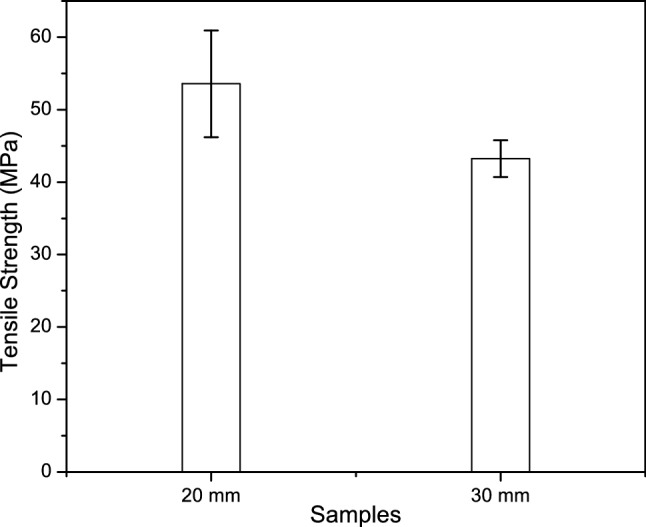
Tensile strength curve of CTF fibers.

The tensile tests of CTF fiber were performed to investigate mechanical properties in two different gauge lengths (GL) that are tabulated in Table 2. Four important properties, tensile strength, Young’s modulus, elongation at break, and energy at break were studied in the tensile test, Fig. 5. In this study, Fig. 6 shows the tensile strength of 30 mm GL is significantly lower with respect to 20 mm GL. The tensile properties of CTF fiber are subjected to the mechanism of fiber extraction from fruits, maturity of the fruits, climate condition, microstructure, and fiber flaws that the cracks initiate. In the case of GL (30 mm), the more flaws present and join with each other cause sudden failure. The tensile test results are influenced by the following parameters: gauge length, accuracy of the instrument, grips, and compliance of the tensile strength testing machine. The Young’s modulus is determined in the elastic zone of the stress–strain curve at each sample GL. The GL of CTF fiber does not affect modulus significantly.

**Table 2:** Summary of tensile characteristics of CTF single fibers.

Gauge length (mm)	Diameter (mm)	Tensile strength (MPa)	Young’s modulus (MPa)	Elongation at break (mm)	Energy at break (J)
20	0.245–0.51	53.55	451.81	17.50	0.05381
30	0.23–0.52	43.24	572.21	18.09	0.08968

**Figure 7:**
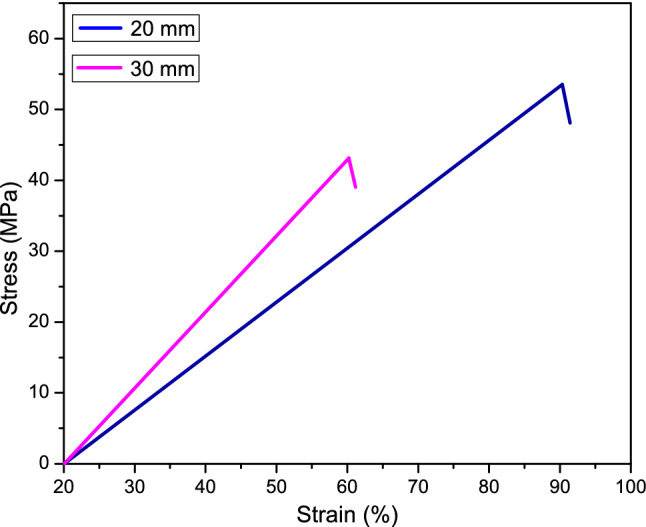
Tensile stress–strain curves of GL 20 mm and GL 30 mm of CTF fibers.

The result shows only 120.40 MPa Young’s modulus higher of 30 mm GL than 20 mm GL. It is expected that Young’s modulus of natural fibers will increase with the increasing GL due to the probability of the arrangement of flaws in terms of volume and size of the fiber. In contrast, the strain to failure will decrease with the increasing GL (Fig. 7). From Fig. 7, it is observed that the strain of 20 mm GL fiber higher than the 30 mm GL fiber. The energy at break depends on the area of elongation at break and the tensile strength of the fiber. The GL 30 mm fiber exhibits the required energy at break 0.08968 J which is 0.03587 J higher from the GL 20 mm. In this experiment, it can be said that higher the elongation at break sharply increases the energy at break, presented in Table 2.

**Figure 8:**
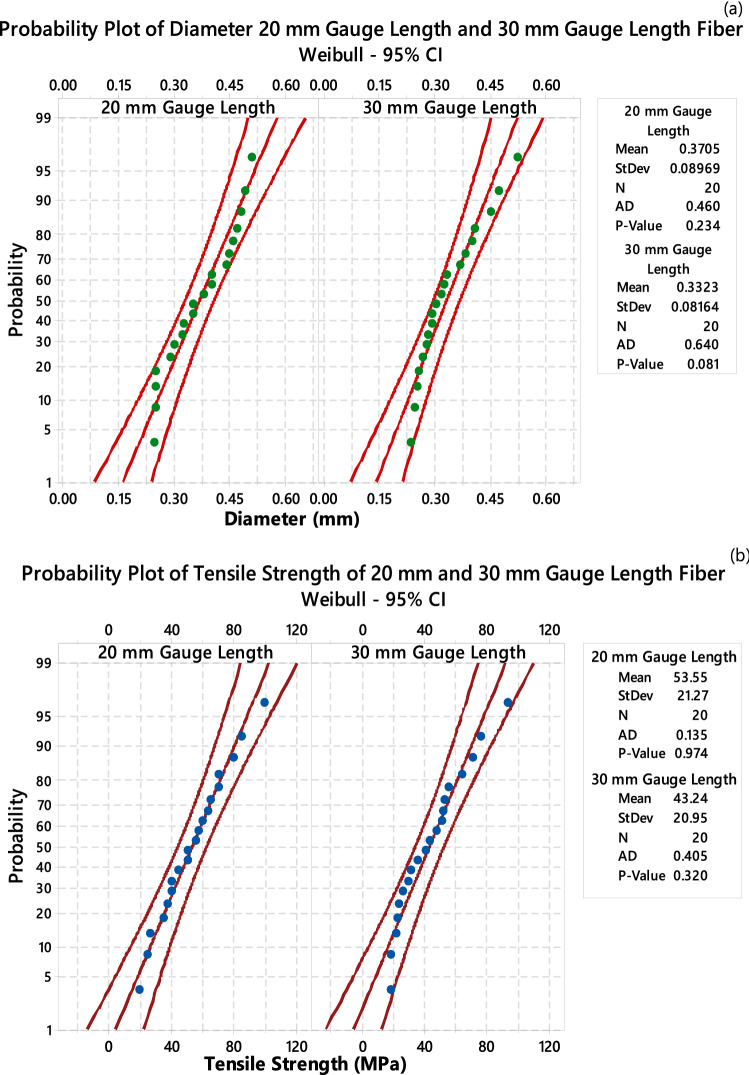
Weibull distribution plot (**a**) diameter and (**b**) tensile strength of CTF fiber.

The Weibull distribution curve analysis of the diameter and tensile strength of CTF fibers were shown in Fig. 8. It can be noticed that the values of diameter and tensile strength are positioned within limits and fit perfectly to the Weibull distributions. From this analysis, it can conclude that the two parameter’s Weibull distribution provides the mechanical properties very close to the experimental mean values.

### Thermal analysis

**Figure 9:**
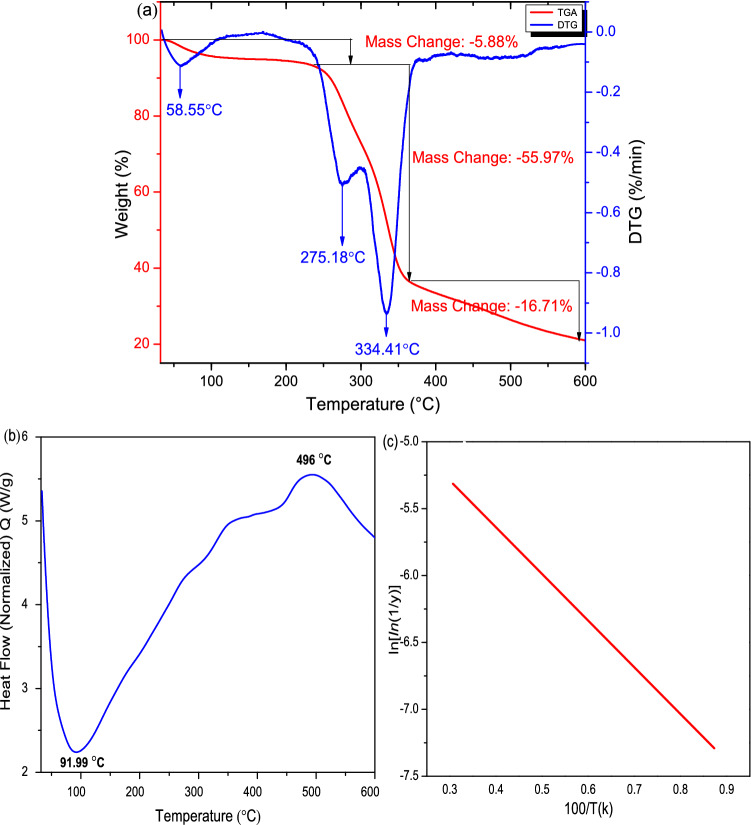
TGA (**a**), DSC (**b**) and Broido’s plots (**c**) of CTF fiber.

The thermal analysis of the CTF fiber was calculated with the help of thermogravimetric analysis (TGA) and derivative thermogravimetry (DTG), differential scanning calorimeter (DSC), and Broido’s plot as shown in Fig. 9a–c, respectively. A natural fiber, when it is exposed to high heat conditions, thermal degradation occurs in the following sequences: hemicellulose, cellulose, lignin, wax and rest of the constituents. The degradation of CTF fiber ingredients was undertaken in three different steps. From figure (a), the initial degradation was occurred at about 100–230 °C. At this temperature, moisture started to evaporate from the CTF fiber. In the second stage of degradation most of cellulose and lignin content is performed degradation, and shows mass loss around of 56% takes place at 365 °C. The highest degradation was obtained with a shoulder at around 334.5 °C and 275 °C, respectively. The last stage of degradation, the cellulose degradation occurs from (366–530 °C) which coincides to the degradation of lignin as well as wax which leaves ash as residue. Moreover, the thermal stability of the CTF fibers to be comparatively resembles than that of *Tridax procumbens*^9^. The energy required to start the CTF fiber degradation is called kinetic activation energy (Ea) presented in Broido’s plot (Fig. 9c) and the required energy is 76 kJ/mol. The TGA analysis elicits that the CTF fibers are an appropriate substance to be performed as a better reinforcement for industrial application.

### SEM analysis

**Figure 10:**
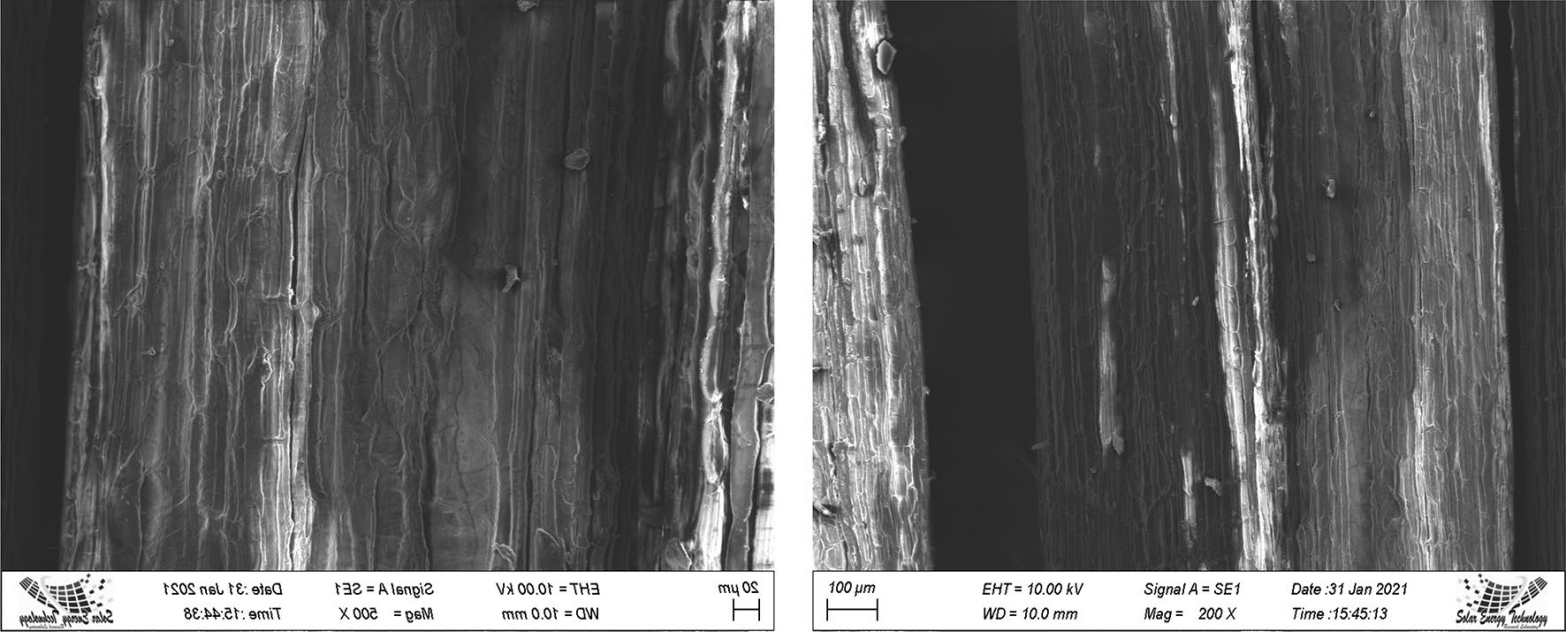
SEM micrographs of CTF fiber.

The surface morphology analysis of the CTF fibers was carried out using SEM. In Fig. 10, the surface is irregular and contains organic elements and impurities. It can be observed from the images, the fiber was round-shaped and full of microfibrils along the longitudinal surface of the fiber^60^. It was conjectured that the surface contains waxes, dirt, and oils. The surface of the fiber must be suchlike that it reflects good interfacial adhesion with polymer matrix. For the preparation of polymer matrix, the impurities and organic materials must be removed from the CTF fibers by chemical treatment.

### EDX analysis

**Figure 11:**
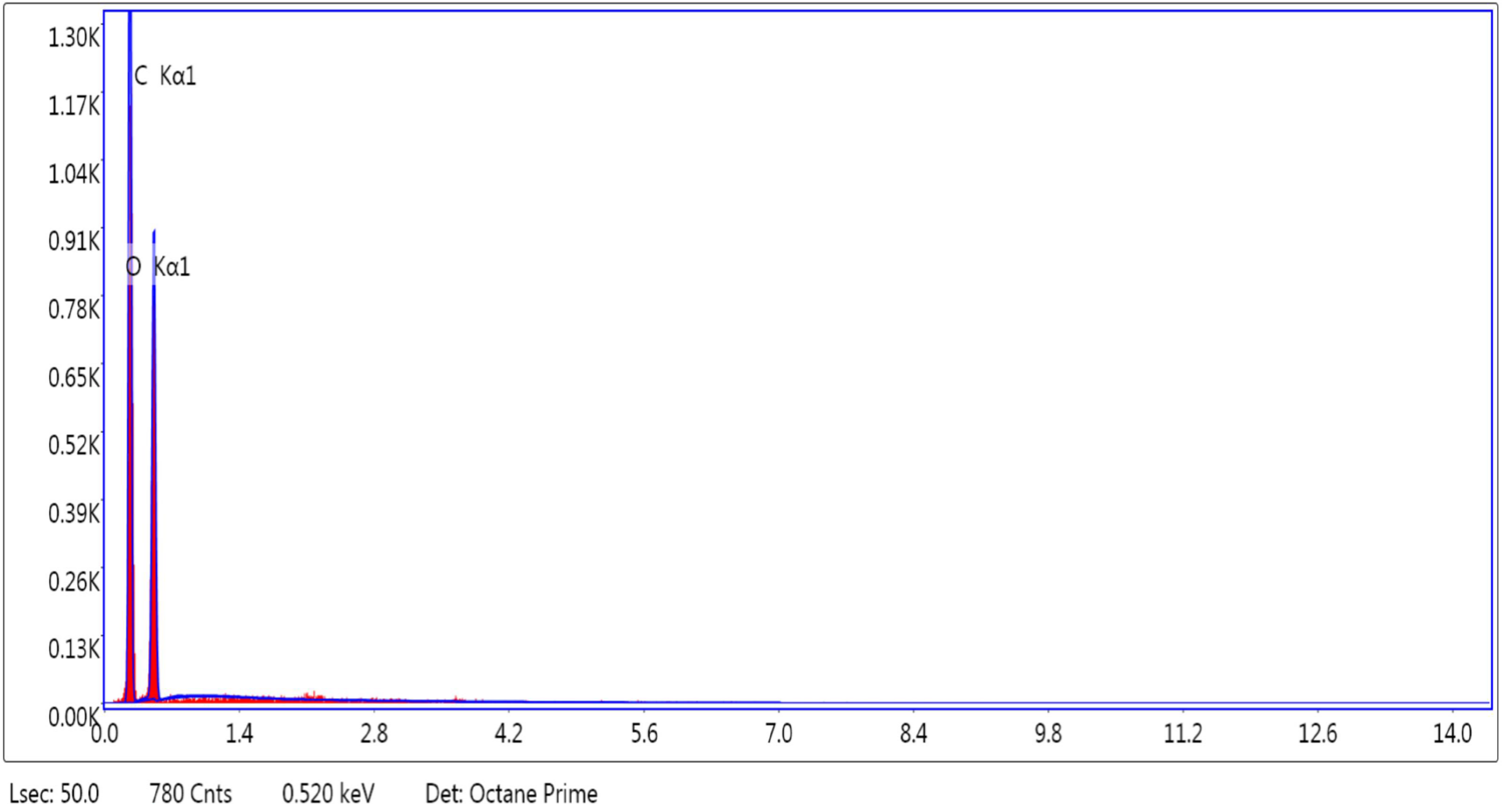
EDX results of CTF fiber.

Figure 11 represents the quantitative elemental analysis of the CTF fiber in terms of weight and atomic percentage. The basic elements in the CTF fibers are carbon and oxygen, which are the major elements on the fiber surface. The weight and atomic percentages of carbon are 55.10 and 62.05, and oxygen are 44.90 and 37.95 respectively.

## Conclusions

This investigation presented the chemical composition, mechanical properties, physical characteristics, thermal, and morphological analyses were studied. The comparatively high percentage of cellulose (55.1 wt%) in CTF fibers can make higher strength and lower density would support them for relatively lightweight composite materials. The average diameter of CTF fiber was calculated to be 131 $\upmu$m. The crystallinity index of CTFs was determined to be 62.5% that shows the high crystalline cellulose. The thermal analysis presented that the stability of the fiber up to 230 °C. The FTIR and EDX analysis of CTF fibers demonstrated the functional group and elements of chemicals. Overall, this investigation advances the facility to work the CTF fibers for different applications, for example, reinforcements in composite and evaluation of physical, mechanical, and chemical properties and CTF fibers suitable sustainable materials for synthesis nanocellulose that is to be used as a reinforcing agent in polymer composites.
